# Mapping Refrigerant Gases in the New York City Skyline

**DOI:** 10.1038/s41598-017-02390-z

**Published:** 2017-06-01

**Authors:** Masoud Ghandehari, Milad Aghamohamadnia, Gregory Dobler, Andreas Karpf, Kerry Buckland, Jun Qian, Steven Koonin

**Affiliations:** 1 New York University, Tandon School of Engineering, Brooklyn, NY USA; 2 New York University, Center for Urban Science and Progress, Brooklyn, NY USA; 3 The Aerospace Corporation, Los Angeles, California USA

## Abstract

Cities are now home to more than 50% of the world’s population and emit large quantities of pollutants from sources such as fossil fuel combustion and the leakage of refrigerants. We demonstrate the utility of persistent synoptic longwave hyperspectral imaging to study the ongoing leakage of refrigerant gases in New York City, compounds that either deplete the stratosphere ozone or have significant global warming potential. In contrast to current monitoring programs that are based on country-level reporting or aggregate measures of emissions, we present the identification of gaseous plumes with high spatial and temporal granularity in real-time over the skyline of Manhattan. The reported data highlights the emission of chemicals scheduled for phase-out. Our goal is to contribute to better understanding of the composition, sources, concentration, prevalence and patterns of emissions for the purposes of both research and policy.

## Introduction

Cities are home to the majority of the world’s population, and as a result, have an outsized impact on the environment. The high urban population density, industrial activity and supporting infrastructure lead to the emission of significant quantities of pollutants into the atmosphere. For example, the burning of fossil fuels results in emission of Nitrogen Dioxide (NO_2_), Ozone (O_3_), Sulphur Dioxide (SO_2_), and Carbon Dioxide (CO_2_), while the leakage of refrigerants from commercial, domestic, and portable refrigeration systems results in the emission of large amounts of Chlorofluorocarbons (CFCs), Hydrochlorofluorocarbons (HCFCs), Hydrofluorocarbons (HFCs), and Ammonia. The detrimental influences of these pollutants on health and environment are well-known and have led to national regulations such as the Unites States’ National Ambient Air Quality Standards^1^ and the European Union’s Directive on Air Quality and Cleaner Air for Europe^2^; as well as international agreements such as the Montreal and Kyoto Protocols^3, 4^, respectively celebrating their 30^th^ and 20^th^ anniversaries in 2017. Monitoring of refrigerant gas emissions, however, is based on self-reported country-level inventories or on aggregate measurements from moving airborne platforms. The limited spatial and temporal resolution of these monitoring techniques makes it difficult to arrive at a comprehensive understanding of these emissions and to promote compliance through the identification of individual sources.

The detection of refrigerant gases is of particular interest due to their environmental impact. Chlorofluorocarbons (CFCs) served as the primary coolants in domestic and commercial air conditioning and refrigeration during the 20^th^ century, and were identified as the primary contributor to the depletion of stratospheric ozone. As a result, the 1987 Montreal Protocol imposed a series of increasingly stringent limits on the production and use of CFCs, leading to a complete ban by 2010. The interim replacement gases for CFCs were Hydrochlorofluorocarbons, (HCFCs), which are also ozone depleting substances but with weaker ozone depletion potentials (ODPs)^5^. The current planned replacement for both of these compounds are Hydrofluorocarbons, (HFCs) with far lower ODP. The near elimination of CFC’s has thus led to a dramatic increase in the concentration of HCFCs and HFCs^5^. While these steps alleviated the threat to the Ozone layer, CFCs, HFCs and HCFCs are also very powerful greenhouse gases (with global warming potentials as much as 10^4^ times greater than CO_2_), so that the steep increase in their concentrations somewhat offsets the mitigation of anthropogenic radiative forcing, now being sought largely through the reduction of CO_2_ emissions^6^.

Our current understanding of the sources and increase in emissions of leaked refrigerant gases derives from two data sources: ground-based and airborne point measurements, or from reports of the quantity of products manufactured and purchased by parties to the Montreal Protocol^5^. Both approaches have been crucial to the science and policies designed to limit ozone depleting substances. However, the measurements refer only to aggregated amounts of release, without a spatial specificity, and the inventory-based methods only identify potential risk and levels of emissions, not actual releases. Accurate monitoring strategies that directly reveal source location, timing, nature and quantities of emissions (e.g., at building-level resolution), would provide an additional layer of data on emissions, addressing the ongoing and increasing use of refrigerants.

Cities inherently have a large number of emission sources and therefore can serve as useful grounds for better understanding of the level and mechanisms of refrigerant leaks. Imaging spectroscopy is particularly well-suited for this phenomenology. Depending on the size of the region of interest and vantage point of the sensor, an instrument can image nearly an entire city with the necessary cadence. Hyperspectral sensors are capable of recording images in hundreds of spectral bands, allowing the determination of the chemical and physical properties of the objects in the scene, including the airspace^7–9^. These instruments offer a combination of a wide field of view and meter-scale spatial resolution which enables the detection, characterization, and tracking of plumes and their sources in near real-time.

Hyperspectral imaging (HSI), similar to the one we have used in this study, has found numerous applications in atmospheric sensing, including plume detection, airborne imagery, and radiative transfer modeling^10–13^. HSI sensors are typically deployed in a “downward-looking” configuration, mounted on moving platforms such as aircraft and satellites. For example^14^, airborne hyperspectral imagery has been used to investigate the emission of ammonia from fumarolic vents in the Salton Sea Geothermal Field, California, measuring the plume concentration and flow rate.

In contrast to airborne observations, stationary ground based imaging offers the benefit of persistence. This is an asset for investigating phenomenology that is otherwise unavailable in aerial remote sensing. A horizontally mounted hyperspectral imager located at an urban vantage point can identify, track and characterize plumes of specific pollutants as they are emitted from hundreds of individual structures.

Following some of the pioneering work in ground based urban sensing^15^, we demonstrate the monitoring capabilities of a horizontally mounted hyperspectral imager by observing the release and subsequent plume of an inert chemical tracer (1, 1-Difluoroethane). We then illustrate the application of the technique by monitoring the persistent leakage of refrigerant gases from a large number of structures in an eight kilometer swath of the Manhattan skyline.

### Quantitative Analysis of Gaseous Plumes

**Figure 1:**
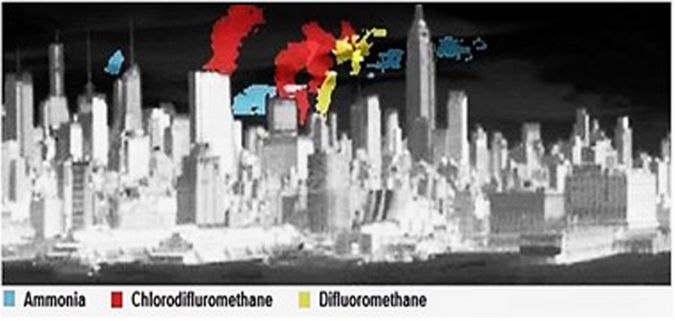
Non-simultaneous composite of compounds detected over Midtown Manhattan. Data cubes post-processed using HyperSEAL software (Aerospace Corp).

**Figure 2:**
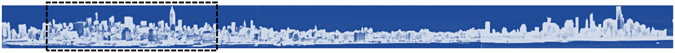
Eight kilometers along Manhattan West side was scanned for eight days at approximately 3 minute intervals. (image by python https://codeshare.io/5ONkBP script, one wavelength from data cube) Results of analysis presented refer primarily to the portion shown above within the dashed line, extending approximately from 14^th^ to 60^th^ street.

We carried out an 8-day imaging campaign in April, 2015 using a ground based, long-wave infrared (LWIR) HSI sensor. The device was deployed on a rooftop observatory in Hoboken New Jersey, with an unobstructed view of the West Side of Manhattan (Figs 1 and 2). That vantage point allowed us to observe a diverse urban scene, ranging from low rise residential structures to high rise buildings. Manhattan’s Westside was scanned at cadences ranging from 10 seconds to 3 minutes, yielding detailed views of the urban landscape in space, time, and radiance spectrum.

**Figure 3:**
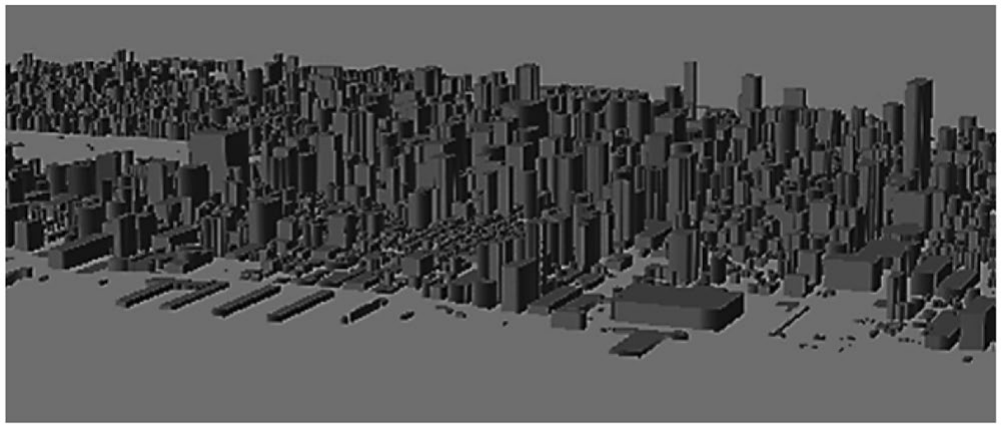
3D digital surface model of Manhattan used for atmospheric correction and cross referencing of remote sensing with correlative data. (image using ARCGIS).

The imaging system employed was the GBSS-2 manufactured by the Aerospace Corporation. Post processing was done using two independent methods, HyperSEAL software provided by Aerospace for plume identification, as well as an analysis procedure using MODTRAN 12 together with a Digital Surface Model (Fig. 3) of NYC for atmospheric compensation and concentrated analysis. The instrument has been in use for several decades. Its spectral range (7.6–13.2 μm) covers a region in which many polyatomic molecules have well-defined spectral features, and the high spectral resolution (40 nm) allows the compounds to be identified with high selectivity. A key factor in the choice of the instrument was its sensitivity. The Noise Equivalent Spectral Radiance (NESR) of ~1 μFlick enables compound identification even when there is low temperature contrast between the plume and background objects (*e.g*., low concentrations of the molecule of interest). This spectral range also corresponded to the peaks of blackbody spectra for temperatures between 220 K and 380 K, thereby allowing for the estimation of solid surface temperatures; this is necessary for calculating the atmospheric correction to the sensor detected radiance for concentration calculations. The 1.1 mrad angular resolution of the instrument applied to target distances ranging from 1 to 5 km, corresponds to a spatial resolution of 1.1–5.5 m per pixel, depending upon distance. Compared to other types of longwave hyperspectral imagers, such as those based on Fourier Transform Infrared (FTIR) Spectroscopy, the system used in the reported work has significantly higher radiometric sensitivity and greater simultaneous spatial coverage. Comparable FTIR based instruments^16, 17^, have smaller spectral coverage (7.7–11.8 μm), thereby limiting the number of identifiable chemical species. Additionally, the NESR (Noise Equivalent Spectral Radiance) and of most FTIR instruments is lower.

**Figure 4:**
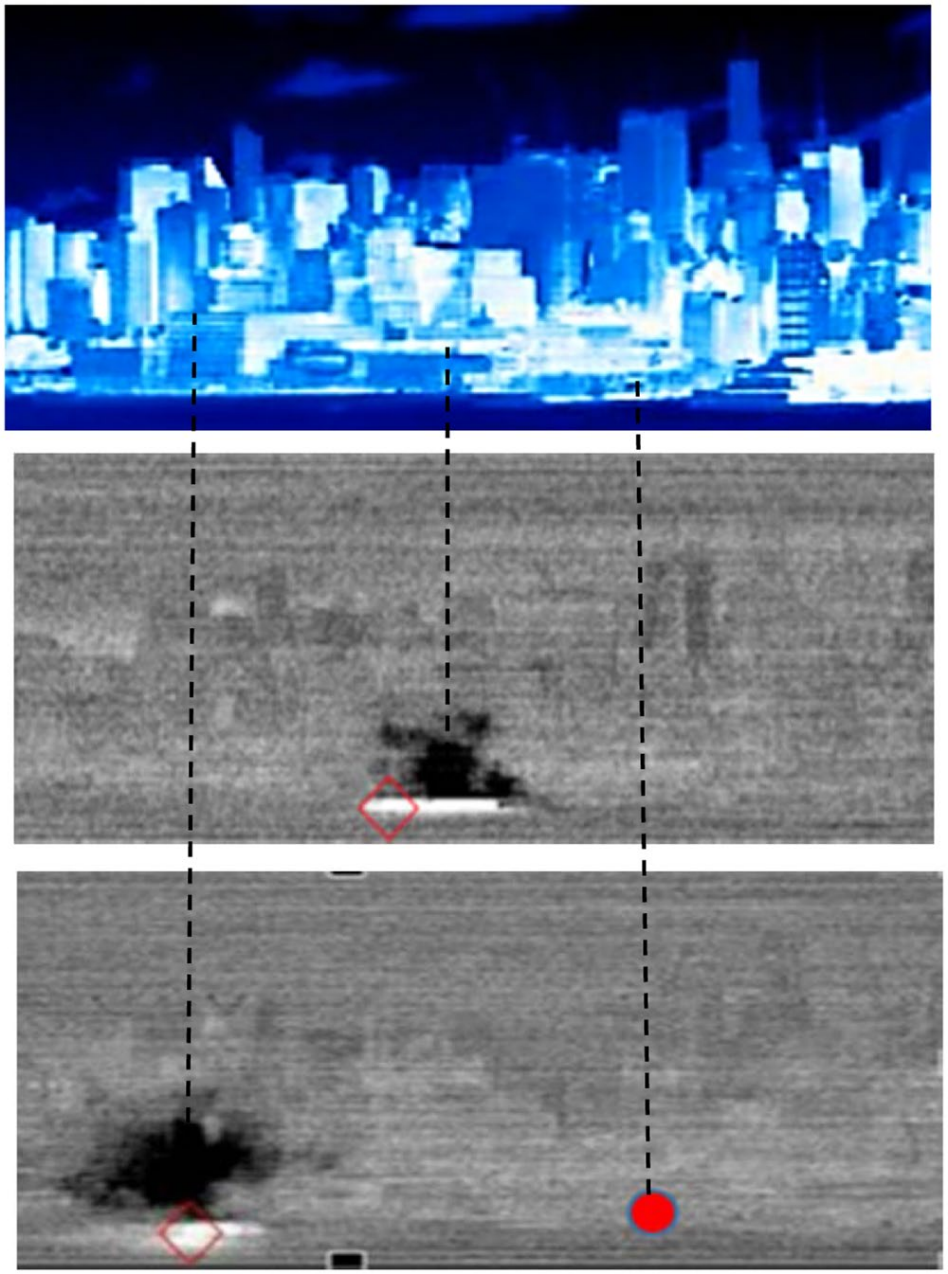
Controlled release test using a canister of DustOff. The upper portion shows false-color depiction of the scene corresponding to measured radiance at 8.2 µm. The lower images show the matched filter intensity profile of the plumes (obtained using HyperSEA software (Aerospace Corp) traveling at average velocity of 8.6 kph at 65 m and 145 m from the point of release, shown by red circle. Red diamonds point to the pixel with maximum signal, which in this case is the thermal reflection of the plume on the river.

We observed a controlled release of a benign chemical to test the instrument and the general methodology, and in particular, the accuracy of the compound identification (ID) algorithm and instrument sensitivity. We released approximately 200 grams of an inert gas (1, 1-Difluoroethane, brand name: DustOff^TM^) at Chelsea piers on the Westside of Manhattan. The gas was allowed to disperse under ambient conditions (the wind blowing from South to North at approximately 2 m/s), while the resulting plume was imaged at a 3-second cadence. Figure 4 shows the point of release (in red) and the plume (in black). The images shown correspond to the plume traveling at an average of 2.4 m/s, when at 65 m and 145 m from the point of release.

**Figure 5:**
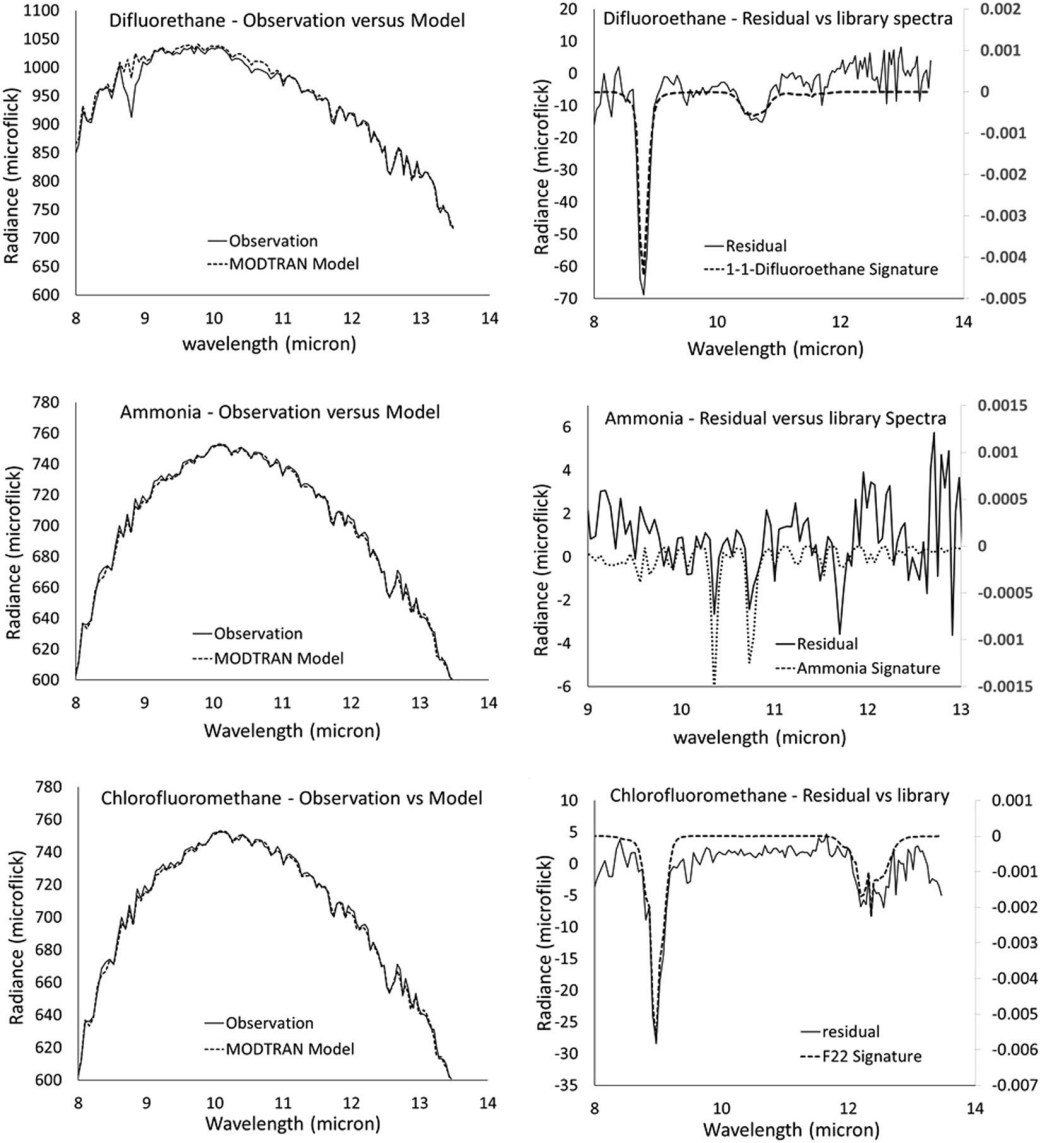
Top (control release, Difluoroethane), Middle (Ammonia), Bottom (Chlorodifluoromethane). (Left) Measured versus modeled path radiance, (Right) Library spectra versus residual calculated after correction for atmosphere, and removal of estimated surface temperature. It is important to note the presence of strong water absorption lines at 11.7 and 12.9 microns, as well as weaker water lines at 11.9, 12,1, 12.3 and 12.5 microns in the Ammonia residuals versus library spectra plot.

The measured long wave radiance includes gray body emissions from the background, scattering, absorption or emission features associated with the ambient gases along the path of observation, and radiance from the target plume. To arrive at a measure of the plume concentration, atmospheric effects were modeled and compared with the measured spectral radiance (Fig. 5). We used the Moderate Resolution Atmospheric Transmission (MODTRAN) model incorporating the radiometric effects from the sky (*e.g*., reflection) as well as the prevailing atmospheric effects in an oblique view of the scene^12, 18^. The model had twenty-eight input parameters. Twenty-five of these were the atmospheric concentrations of various gases, twenty-two of which were taken from conditions typical in a mid-latitude summer air column (see Table 1), while (H_2_O, O_3_, CO_2_) concentrations were measured ambient values obtained from local weather stations (Table 2). The remaining three parameters were air temperature, background surface temperature, and emissivity. Air temperature was obtained from local weather stations records. The surface temperature and emissivity of a background solid surface behind a plume was calculated using an iterative process in the MODTRAN. This process modeled the background based on observations made of that same position in the scene after the plume had passed by. In addition, the determination of the background surface temperature required knowing the path length from sensor to each corresponding building surface. This was obtained using a 3D digital surface model of the city (Fig. 3) derived from the NYC building data^19^. The values above were used as input to MODTRAN for the atmospheric corrections using the gradient descent approach^12^. This information, along with data on the surface topology of the physical infrastructure in NYC, was used to derive the column spectra. Specifically, we used the Adaptive Coherence Estimator (ACE)^20, 21^ for detection; the filter outputs were then automatically inspected to provide ROI’s (regions of interest) and each of the ROI’s were subsequently ID’d using stepwise GLS in whitened space.

**Table 1:** Concentration of trace gases used for atmospheric correction.

Compound	Concentration (g/cm^2^.m)
F11	2.93E-11
F12	5.03E-11
CCL3F	2.10E-19
CF4	2.10E-19
F22	1.26E-11
F113	3.98E-12
F114	2.51E-12
R115	2.10E-19
CLONO2	1.21E-12
HNO4	9.10E-14
CHCL2F	2.10E-19
CCL4	2.72E-11
N2O5	5.08E-17
CO	3.14E-08
CH4	3.56E-07
N2O	6.71E-08
O2	4.38E-02
NH3	1.05E-10
NO	6.29E-11
NO2	4.82E-12
SO2	6.29E-11
HNO3	1.05E-11

**Table 2:** Measured values used for Atmospheric compensation.

	Difluorethane	Ammonia	Chlorodifluoromethane
Timestamp	04/13 15:22 EST	04/08 09:11 EST	04/08 09:11 EST
Water vapor	1.47 g/cm^3^	1.50 g/cm^3^	1.50 g/cm^3^
Ozone	0.00026 g/cm^3^	0.00064 g/cm^3^	0.00064 g/cm^3^
Carbon Dioxide	400 ppmv	400 ppmv	400 ppmv

Figure 5 shows, for each of three targeted gases (Difluoroethane, Ammonia, and Chlorodifluoromethane), two sets of spectra: the MODTRAN modeled fit and the recorded spectra (left panels), and the residual spectra (i.e., the spectra after the blackbody radiance is removed and the atmospheric correction was applied) along with the library spectrum for the corresponding compound (right panels). The plots on the left highlight the blackbody spectrum as well as absorption and emission features of the target gases. The residuals and library spectra are used to identify the compounds in the corresponding horizontal column of air.

The Instrument deployed has exceptionally low NESR (Noise Equivalent Spectral Radiance), one which is periodically calculated during the pre- and post- target calibrations. For example an NESR of 0.7–1.0 microflicks was obtained in many of the detects. The MDQ (Minimum Detectable Quantity) is a function of the NESR, as well as processing methodology (e.g., atmospheric correction) and compound characteristics (e.g., depth of absorption line and wavelength of peak absorption). The MDQ can be calculated by taking the ratio of the concentration of a particular sample (obtained using the strength of the absorption line, the absorption cross section and the plume geometry) and its signal-to-noise ratio (SNR). Here the SNR is found by taking the ratio of the depth of the absorption line (see Fig. 5) and the standard deviation of the signal in a region immediately adjacent to the absorption feature (where no molecular absorption occurs).

The signal-to-noise ratios for 1–1-Difluoroethane, Ammonia and Chlorodifluoromethane, were 27.6, 2.0 and 17.9, respectively (see Table 3). This corresponds to a sensitivity of detection (i.e., MDQ) of 0.06 ppm for 1-1-Difluoroethane, 0.25 ppm for Ammonia, and 0.06 ppm for Chlorodifluoromethane. These calculations used the background surface temperature, and assumed plume depth to be the same as plume width.

**Table 3:** Minimum Detectable Quantities of Target Species.

Compound	Concentration (ppm)	Signal (microflicks)	Noise (microflicks)	Calculated SNR	MDQ (ppm)
1-1-Difluoroethane	1.7	65.2	2.4	27.6	0.06
Ammonia	0.5	1.92	0.96	2.0	0.25
Chlorodifluoromethane	1.0	27.4	1.5	17.9	0.06

**Figure 6:**
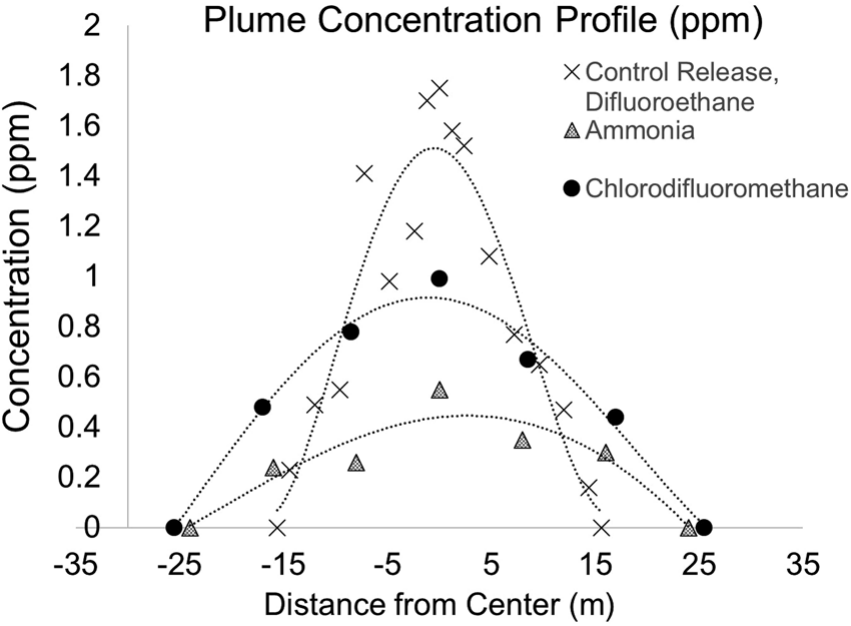
Estimation of plume concentration based on column density spectra. The concentration profiles shown in this Figure are for horizontal slices through the centers of the plumes seen in Fig. 4 (frame 2) and Fig. 7.

Figure 6 shows the estimated plume concentration profile along a horizontal line through the selected plume centroid (see supplementary document for details). The figure shows a maximum Difluoroethane concentration of 1.7 ppm for the 30-meter-wide plume located 65 meters from the point of release. We calculated the expected concentration of Difluoroethane to be 1.2 ppm, based upon the dispersion of the known quantity of gas in the Dustoff^TM^ canister, the assumption of an axially symmetric Gaussian concentration profile, and the known absorption cross section of the compound^22^. During this controlled release experiment, the wind velocity was approximately 2.4 m/s due North. This was measured by observing the plume centroid move 80 m in 33.5 seconds (as shown in Fig. 6 frames 2 and 3). This agrees with records of meteorological conditions. The transverse dispersion rate was assumed to be the same as the vertical dispersion rate (0.4 m/s obtained by observing the plume expand from a height of 20 m to 30 m in 33.5 sec.) The difference between the measured and calculated concentrations is in part due to this symmetry assumption.

**Figure 7:**
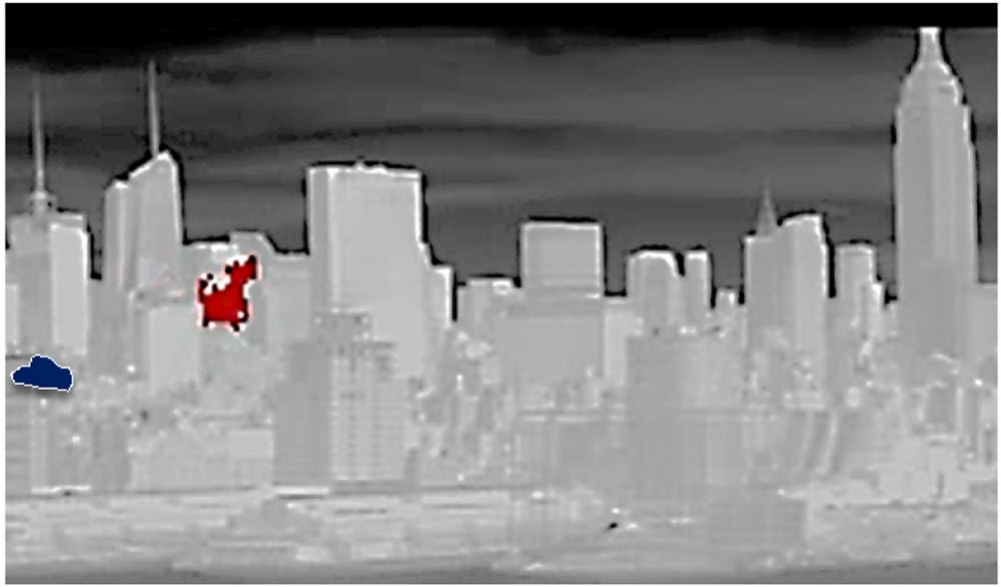
Selected plume for further analysis: (dark red) Chlorodifluoromethane, (dark blue) Ammonia. [Plumes obtained using HyperSEAL software (Aerospace Corp).

Figure 6 also shows the estimated plume concentration profiles of selected Ammonia and Chlorodifluoromethane plumes in Midtown Manhattan on April 12 (Fig. 7 identifies the specific plumes analyzed). The distances to the plumes were estimated using their proximity to specific buildings in the field of view. The Ammonia plume and the Chlorodifluoromethane (HCFC-22) plume, each approximately 50 m wide, have maximum concentrations of 0.5 ppm and 1.0 ppm, respectively, compared to the approximate 10 ppb ambient urban concentration^23, 24^. The calculations used the same atmospheric correction approach described above, as well as the assumption of an axially symmetric Gaussian plume profile.

**Figure 8:**
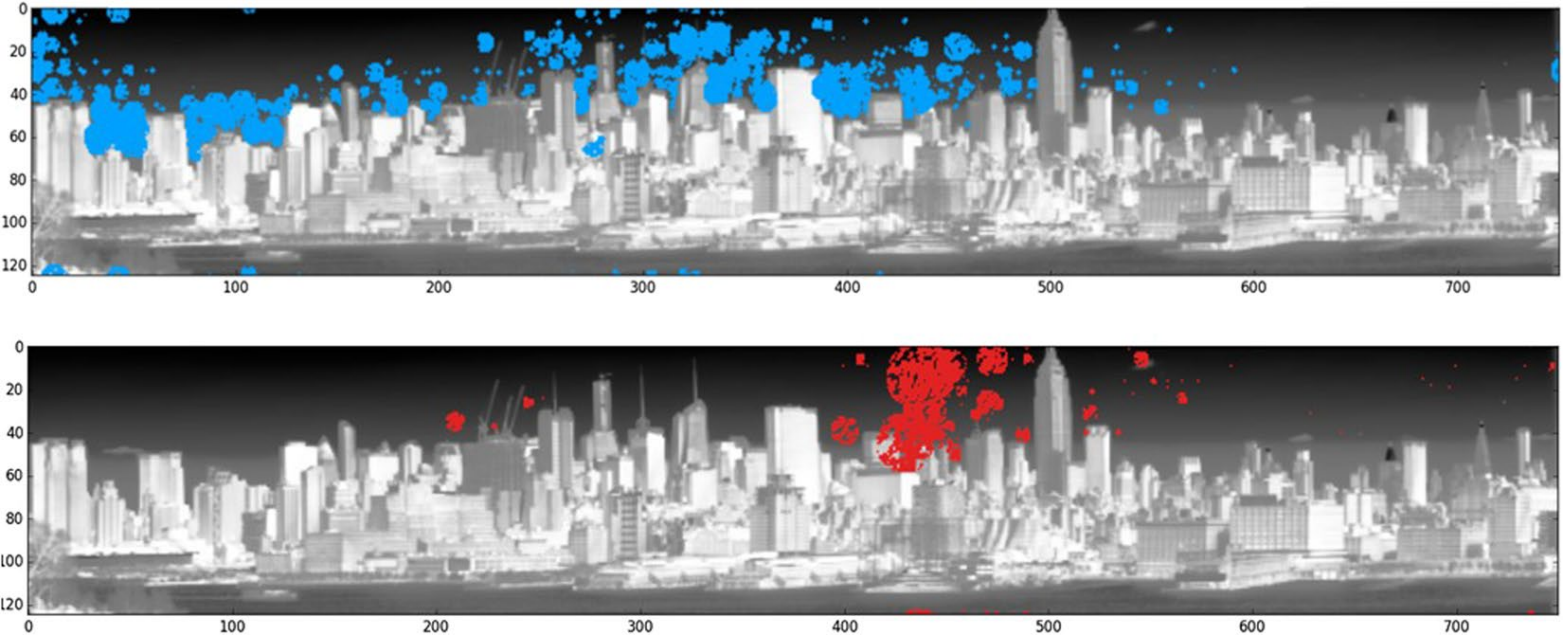
Plume distribution accumulated in Midtown Manhattan in April 12, 2015: (top) Ammonia, (bottom) Chlorodifluoromethane (Freon22). [Plume pixel ID by HyperSEAL software (Aerospace Corp). and by Python coding https://codeshare.io/5ONkBP].

It is important to note that identifying the exact location of a plume can be challenging when using only one instrument as was done in the proof-of-concept work reported here. We were able to estimate the location of certain plumes when they were seen either between buildings (as in the case of the red-highlighted Chlordiofluoromethane plume in Fig. 7) or in front of a particular background building (here we estimate that the plume is over Manhattan, but in front of the particular building). In the case of plumes in the sky (as seen in Fig. 8), we may assume that they are over the island of Manhattan (but we acknowledge that there is uncertainty in the exact distance). Deployment of two HSI sensors will allow the identification of plume locations and/or sources.

We have accounted for the effects of component gases in the horizontal column of ambient air between the sensor and the plumes in two ways. First, as described earlier, an atmospheric correction was applied based on meteorological conditions at the time of the observations and the use of MODTRAN. Second, we needed to account for the possibility of diffuse, low concentrations of the target gas species present in the horizontal column. This was accomplished by analyzing the spectra from horizontal columns from the imager to locations in the scene that were separate from, but near the plumes. These columns partially overlapped with the horizontal columns leading to the plumes, with the remaining portions being spatially close enough that one can readily assume that they contained the same atmospheric constituents. The residual spectra were generated for these columns after applying the same atmospheric correction used for the plumes. Results did not display any significant background concentration. As a result, one can conclude (to within the detection sensitivity of the instrument) that there was no significant interference to our plume measurements due to diffuse, low concentrations of the target gas species in the intervening ambient air.

We have validated the detection scheme in a number of other applications in a downward-looking geometry, using several sets of ground truth data. This included a series of “blind collections” in which the “truth” was not known until after the results were calculated^25, 26^. Our ID algorithm makes use of a library containing the spectra of approximately 800 chemicals. In the case of a mixture, the ID algorithm results in a combination of chemicals (and coefficients) whose combined spectra represents a best fit to the data even in the presence of multiple gases^27^. The ID algorithm relied on matching the relative location and intensity of multiple peaks, rather than the intensity of the principle peak. After the pixels are identified, a prescribed T-statistic value was used to approximate the plume boundaries; the column density spectra were then determined in real space.

The study identified 10 different types of gaseous plumes including HCFCs and HFCs, and Ammonia (see Table 4). Figure 8 shows a cumulative map of Ammonia and Chlorodifluoromethane (HCFC-22) detected in one day; the plume maps are composites of plumes detected for each compound over 24 hours on April 11, 2015. The map was obtained using data from scans of the scene at a cadence of approximately 3 minutes. These observations include plumes detected at multiple times but may be originating from the same source. On average, each data cube, representing three minutes in time, contained two plumes of a gas used primarily as coolants for buildings and appliances^28^. This rate of observed HCFC plume production is quite high considering the U.S. EPA’s goal of completely eliminating their use by 2020.

**Table 4:** Detected Gases.

Chemical Designation	Name
NH_3_	Ammonia
HCFC-22	Chlorodifluoromethane
CO_2_	Carbon Dioxide
HFC-32	Difluoromethane
R-134a	1,1,1,2-Tetrafluoroethane
CF_3_CHF_2_	Pentafluoroethane
CH_4_	Methane
SO_2_	Sulfur Dioxide
R-143a	1,1,1-Trifluoroethane

During the course of the study, we observed an order of magnitude greater number of HCFC-22 plumes than 1,1,1,2-Tetrafluoroethane (R-134a) plumes; specifically, 1722 HCFC-122 plumes were identified, while only 197 plumes of R-134a were observed. R-134a, an HFC, is the preferred replacement for CFCs in motor vehicle air conditioning units, and has a relatively high global warming potential (1300 times that of CO_2_)^5^. The growing prevalence of these gasses has been reported by Xiang, *et al*.^5^. It should be emphasized that the U.S. EPA regulations had mandated drastic reduction of HCFC emissions (i.e., HCFC-22) by 2015; therefore, the detection of excessive emissions of HCFC was a surprise. In the case of R-134a, agreements for the reduction of R-134a adopted in October of 2016 via the Kigali Amendment to the Montreal Protocol^24^) is expected to result in significant reduction of R-134a. It should be added that R-134a is primarily emitted at street level, while HCFC-22 is emitted from large chillers typically placed on the roofs of buildings. This may introduce a bias in our observations since the detected plumes were at or above the roof level of the surrounding buildings.

An additional unexpected observation was the prevalence of Ammonia in the NYC atmosphere. Sources of Ammonia in the Metropolitan New York Atmosphere include on-road mobile sources, composting, industrial refrigeration, and aspiration of sewage emissions from large building stacks^27^. It is worth noting that Ammonia, as a refrigerant, is reemerging in industrial and commercial use as a replacement for HCFCs. Ammonia is cost effective and causes no ozone depletion. Although it is not a greenhouse gas, there are concerns with its use. For example, Ammonia is toxic at concentrations above 30 ppm and is flammable at concentrations above 15% in ambient air (compared to 5% for Methane). It is highly reactive with copper, commonly used in CFC based piping. While the copper infrastructure in developed countries has transitioned toward stainless steel, such an expense for underdeveloped nations has been an obstacle to the use of Ammonia. Considering a variety of factors including accidents resulting from poorly maintained facilities, handling by untrained individuals, and potential use in terrorism, its use has come under increased scrutiny. Ammonia emissions are of particular concern due to their formation of fine aerosols through the creation of ammonium salts such as ammonium sulfate ((NH_4_)_2_SO_4_) and ammonium nitrate (NH_4_NO_3_). This particulate matter has a significant effect on the Earth’s radiation budget as well as adverse health effects on the human population^29^. In future work, we will correlate the plume findings with registration/permits for large cooling towers and scrubbers, to gain an understanding of the nature of these emissions.

## Conclusions

We have shown the potential of persistent ground based long-wave hyperspectral imaging to monitor refrigerant gases leaking from a large number of structures in an 8-km swath of Manhattan in real time. These include large quantities of Chlorodifluoromethane (HCFC-22) and Ammonia along with a number of other gases. Considerable quantities of HCFC-22 plumes were detected even though the United States is phasing out its use by 2020. Another unexpected observation was the prevalence of Ammonia in the NYC atmosphere. The oblique view imagery, the digital surface model of NYC, and the MODTRAN atmospheric model were used for estimation of plume concentrations. A controlled release was carried out to test the proposed approach by comparing the concentration derived from the imagery versus that obtained using the known quantity of a plume of an inert chemical (1, 1-Difluoroethane). Our results suggest that long-term spectroscopic imaging campaigns will be instrumental for estimating gaseous refrigerant emissions for the purpose of both research and policy, and are especially practical in population centers. Follow up studies will include searches for other atmospheric and trace gases in the spectral library as well as the deployment of two imagers for enhanced localization and trajectory information. The latter will allow us to correlate plume dynamics with the multitude of data on NYC’s infrastructure and operations including information on fuel type and HVAC infrastructure.

## Methods

The hyperspectral imager reported in this work was deployed on a fixed platform with an oblique view of the NYC skyline. Prior to this study the system has been used for gas detection from airborne platforms. Supplementary details on the sensor calibration methodology, as well as the process for atmospheric compensation and quantitative analysis are given below.

The sensor was calibrated radiometrically by observing two onboard blackbody sources that are stabilized at temperatures spanning the expected radiance of the scene. The blackbody sources are observed immediately before and after scan. A linear relation between the known blackbody input radiance and the digital count output is assumed, and the sensor responses are modeled with gain and offset terms. These terms for the pre- and post-collection calibrations are then time interpolated to match the actual collection time of the data. Sensor wavelength calibration was done by observing the blackbody sources when covered by the NIST traceable transparent polymer films, with calibrated absorption features followed by a least-squares fit across the focal plane array.

A moderate resolution atmospheric transmission (MODTRAN) model^12^ was used to quantify and compensate for atmospheric contributions to the observed radiance, and was customized for oblique view imaging. The output from the MODTRAN model provides the path transmission (*τ*), the radiation (*L*_*u*_) originating along the path of observation (including background surface emission) arriving directly at each sensor pixel, and radiation (L_d_) due to path scattering and radiation from the sky including those reflected from background building surfaces. The model output was subsequently used as input to the radiative transfer equation:

1 $$L(\lambda ,T)=\tau [\varepsilon BB(\lambda ,T)+(1-\varepsilon ){L}_{d}]+{L}_{u}$$

Where *L*(*λ,T*): Radiance reaching the sensor in absence of plume, *λ*: Wavelength, *T*: Surface Temperature, *τ*: Path transmission, *ε*: Emissivity (spectrally invariant), *BB*(*λ,T*): Blackbody radiation, *L*_*d*_: radiation from path scattering, sky and building reflection, *L*_*u*_: radiation originating for the path including building surfaces.

To simplify the solution to the radiative transfer equation, the emissivity was assumed to be wavelength-independent. This may be justified in gas concentration calculations, where the small wavelength dependence of the surface material’s emissivity will have an insignificant effect on the spectral features induced by gases; a stronger variation in the surface emissivity will appear in the residual after atmospheric correction.

The estimation of sky and path scattering radiation (known as down-welling radiation in downward looking imaging) is challenging in the case of an oblique view; however certain assumptions can simplify the calculations. One is the use of similarity between the radiation received by the objects in the scene versus radiation received by the sensor from the sky. The building surfaces in the scene are vertically aligned and receive the sky radiance over a range of 90 degrees (from vertical to horizontal). Meanwhile, the sensor swath width and height are 94 and 6 degrees, respectively. The field of view looking from the instrument at Hoboken toward Manhattan is occupied by approximately 50% sky, 40% buildings and 10% river. The same can be said if the sensor was to be located in Manhattan looking toward Hoboken. Therefore, it can be assumed that the average of all pixels received in the 6 × 94 degree window is representative of what an object receives from its surroundings and radiated toward the sensor. This “in-scene” measurement was used to approximate L_d_.

Difluoroethane and Chlorodifluoromethane are highly detectible, partly due to their large absorption cross-sections and the fact that their absorption peaks do not coincide with the black body radiation peaks typical for ambient temperatures (i,e., in the 9–11 micron region). Ammonia on the other hand, is harder to detect as its absorption peaks overlap the expected peak for ambient black body radiation. Analysis of its spectra are further complicated by its lines’ proximity to the strong water lines at 11.7 and 12.9 microns, and weaker water lines at 11.9, 12.1, 12.3 and 12.5 microns (these water lines are apparent in Fig. 5 in the Ammonia residuals plot at wavelengths longer than 11 microns). As a result, Ammonia is harder to detect when using the intensity of absorption at a single peak. The use of a single peak can make the plume morphology sensitive to variations in background water vapor cloud temperature, resulting in the observed plume shapes following the cloud morphology. However, an analysis looking at features presented by multiple peaks, such as in the reported work, results in improved detection. The HSI instrument was previously used in a downward looking configuration for detection of controlled release Ammonia plumes, and results were very satisfactory^14^.

Using the residual obtained by comparison of atmospheric-corrected, measured values and the library spectra, the concentration of the target molecular species may be calculated using Beer’s law, which is given by:

2 $${L}_{sensor}(\lambda ,T)-{L}_{model}(\lambda ,T)=\alpha (\lambda )\lambda .D.\lambda [BB(\lambda ,{T}_{Surface})-BB(\lambda ,{T}_{Plume})],$$

where *L*(*λ,T*): Radiance (microflick) *λ*: Wavelength (micrometers), *BB*(*λ,T*): Blackbody radiation, *α*: Absorption coefficient, *D*: Path Length, One can use Equation 2 and *α*(*λ*) = *σ*(*λ*)*N* to calculate the concentration:

3 $$N=\frac{[{L}_{sensor}(\lambda ,T)-{L}_{model}(\lambda ,T)]}{\sigma (\lambda )D[BB(\lambda ,{T}_{Surface})-BB(\lambda ,{T}_{Plume})]},$$

where *N* is the concentration.

## Electronic supplementary material

Animation of HCFC Emission

Supplementary Information
