# Supplementary material for: Mapping Refrigerant Gases in the New York City Skyline
Source: Sci Rep. 2017 Jun 1;7:2735. doi: 10.1038/s41598-017-02390-z (PMC5453978; doi:10.1038/s41598-017-02390-z)
Supplement: Supplementary file 2 — Supplementary Information [file 41598_2017_2390_MOESM2_ESM.pdf]

Supplementary Documents

Source of video shown plume emissions

Mapping Refrigerant Gases in the New York City Skyline

Masoud Ghandehari[1], *Milad Aghamohamadnia*[1], *Gregory Dobler*[1], *Andreas Karpf*[1], *Kerry Buckland*[2], *Jun Qian*[2], *Steven Koonin*[1]

[1] New York University, [2] The Aerospace Corporation

Caption: "The video was created by animating multiple plume images into a time resolved depiction of their size and location".

Information for the editor: The images were obtained by post-processing data cubes using software HyperSEAL 7.9.0, developed by the authors, a similar process used for deriving the images shown in figures 1,7, and 8. HyperSEAL is a proprietary product of The Aerospace Corporation, which holds the rights to authorize publication of content produced with that software. http://www.aerospace.org/
For inquiries contact David M Tratt david.m.tratt@aero.org
